# Developmental Markers of Genetic Liability to Autism in Parents: A Longitudinal, Multigenerational Study

**DOI:** 10.1007/s10803-016-2996-x

**Published:** 2017-01-09

**Authors:** Molly Losh, Gary E. Martin, Michelle Lee, Jessica Klusek, John Sideris, Sheila Barron, Thomas Wassink

**Affiliations:** 1 Department of Communication Sciences and Disorders, Northwestern University, Frances Searle, 2240 N Campus Dr., Evanston, IL 60208 USA; 2 Department of Communication Sciences and Disorders, St. John’s University, Staten Island, NY 10301 USA; 3 Department of Communication Sciences and Disorders, University of South Carolina, Columbia, SC 29201 USA; 4 Frank Porter Graham Child Development Institute, University of North Carolina at Chapel Hill, Chapel Hill, NC 27516 USA; 5 College of Education, University of Iowa, Iowa City, IA 52242 USA; 6 Department of Psychiatry, Carver College of Medicine, University of Iowa, Iowa City, IA 52242 USA

**Keywords:** Autism, Genetics, Endophenotype, Longitudinal, Broad autism phenotype, Language

## Abstract

Genetic liability to autism spectrum disorder (ASD) can be expressed in unaffected relatives through subclinical, genetically meaningful traits, or endophenotypes. This study aimed to identify developmental endophenotypes in parents of individuals with ASD by examining parents’ childhood academic development over the school-age period. A cohort of 139 parents of individuals with ASD were studied, along with their children with ASD and 28 controls. Parents’ childhood records in the domains of language, reading, and math were studied from grades K-12. Results indicated that relatively lower performance and slower development of skills (particularly language related skills), and an uneven rate of development across domains predicted ASD endophenotypes in adulthood for parents, and the severity of clinical symptoms in children with ASD. These findings may mark childhood indicators of genetic liability to ASD in parents, that could inform understanding of the subclinical expression of ASD genetic liability.

## Introduction

Autism spectrum disorder (ASD) is a common and complex, genetically-based neurodevelopmental disorder (APA 2013; CDC 2014). ASD is among the most highly heritable of complex psychiatric conditions, with a number of risk loci now identified (Tick et al. 2015). However, the genetic causes of ASD are heterogeneous, with most cases due to polygenic genetic effects, and less than 1% explained by any specific molecular genetic variant. The study of relatives who are at increased genetic liability can help to overcome the challenge of genetic heterogeneity by providing phenotypes that may help to identify biologically meaningful subgroups of families.

ASD is highly familial and subclinical phenotypes that are qualitatively similar to core features of ASD have been repeatedly reported in a subset of relatives, which are believed to constitute endophenotypes (Bolton et al. 1994; Landa et al. 1991, 1992; Losh et al. 2011; Murphy et al. 2000; Piven et al. 1997a, 1994). Endophenotypes are genetically influenced traits existing midstream between disease phenotype and underlying genetics (Gottesman and Gould 2003). They may manifest as a *forme fruste* of complex genetic disorders among unaffected relatives, as in ASD, where relatives often exhibit subclinical personality and language traits similar in quality to the core features of ASD but subtle in expression and not associated with functional impairment. Candidate endophenotypes in relatives of individuals with ASD have been collectively described as constituting a broad autism phenotype (BAP) that may inform etiologic mechanisms by helping to decompose the complex ASD phenotype into more fundamental component features with clearer ties to underlying biology (Losh et al. 2008; Lowe et al. 2015).

Investigations of ASD endophenotypes in parents have, to date, been restricted to assessments in adulthood, when status as a parent of a child with ASD is determined. This prior work has documented elevated rates of personality and language styles in parents that mirror in quality the core features of ASD, including socially reticent/aloof and rigid personality traits, along with differences in pragmatic (i.e., social) language styles (Bolton et al. 1994; Landa et al. 1991, 1992; Losh et al. 2011, 2008; Murphy et al. 2000; Piven et al. 1997a, 1994). Such traits appear to be more strongly expressed among parents from multiplex families than simplex families, suggesting that they are sensitive indices of genetic liability to ASD (Bernier et al. 2011; Losh et al. 2008; Virkud et al. 2009).

Differences among parents have also been observed in neuropsychological domains that are conceptually related to, and have been hypothesized to underlie the clinical-behavioral features of ASD and the subtle personality and language traits of the BAP. For instance, parents of individuals with ASD show differences on social cognitive tasks that tap strategies for contending with complex social-emotional stimuli involving reading thoughts and emotions from faces, the eye region of the face, and from biological motion, (Baron-Cohen et al. 1997; Losh et al. 2009; Losh and Piven 2007) and also exhibit differences in brain activation during such tasks (Baron-Cohen et al. 2006). Differences in language processing skills, indexed by performance on rapid automatized naming (RAN) tasks that tap a broad neural network recruited in complex language skills, have also been observed in both parents and siblings of individuals with ASD, (Losh et al. 2010; Norton and Wolf 2012) as well as reduced language automaticity indexed by eye movement patterns during RAN (Hogan-Brown et al. 2014). Importantly, differences in both social cognition and language processing tasks among parents appear to be driven by the subgroup of parents who show social and pragmatic language features of the BAP (Losh et al. 2009, 2010), suggesting that these personality, language, and neuropsychological phenotypes importantly interrelate and cosegregate in a subgroup of individuals, and may therefore be used to stratify subgroups for further study of the biological underpinnings of the BAP and ASD.

A limitation of this prior work is its focus on adulthood, when it is possible to classify individuals as parents of an individual with ASD. This leaves unexplored a large period of development during which ASD endophenotypes could first arise in parents, and might be most profitably studied for clues into underlying biology and to inform ASD risk in families. Studies of early development in unaffected siblings (Ozonoff et al. 2014) are informative, but knowledge of developmental endophenotypes in parents offer additional value in permitting the tracing of inheritance patterns and characterization of variable manifestation of genetic burden across generations not afforded by affected sibling designs. Moreover, studies of parents during childhood are free from the potentially confounding effects inherent in sibling studies, where it is possible that having a sibling with ASD could impact the content and quality of early language and social experiences (Pilowsky et al. 2007).

This study investigated developmental phenotypes potentially reflecting ASD genetic liability in parents, by making use of archival data from academic testing records over childhood, from a cohort of individuals who as adults would go on to have a child with ASD. Using a retrospective, multigenerational, longitudinal design, we examined development in the domains of language, reading, and math among parents of individuals with ASD from grade school into high school. Parents’ developmental profiles were examined in relationship to putative endophenotypes in adulthood—i.e., personality and language characteristics of the BAP, and social cognitive functioning—as well as clinical symptom severity in their children with ASD. By exploring relationships among candidate endophenotypes within individuals over development, and across generations, we aimed to identify childhood phenotypic profiles in parents that might be used to better understand the developmental origins of the BAP, and intergenerational markers of ASD genetic risk.

## Methods

### Participants

Participants included 139 parents of individuals with ASD, 63 individuals with ASD who were children of participating parents, and 28 controls. Recruitment efforts took place over the course of 5 years throughout the state of Iowa. Recruitment was limited to the state of Iowa because of restrictions on access to archival academic data existing in other states. Families were primarily recruited through the University of Iowa Children’s Hospital Autism Center, the state’s primary comprehensive children’s hospital that serves as the main site for ASD diagnostic evaluations and services in Iowa. A series of recruitment mailings inviting study participation were distributed to families who had visited the clinic for treatment or diagnosis of a child with ASD over the last 20 years. Extensive community-based recruitment efforts were also pursued in both rural and urban areas throughout Iowa to increase sample representativeness. This included recruitment through schools, local clinics, disability advocacy organizations, community events (e.g., advocacy walks and events, community festivals, sporting events, 4-H clubs), and repeated study advertisements through state-wide broadcast radio and print ads.

Families of individuals with ASD were eligible to participate if one or more parents had attended school in Iowa as a child, were native speakers of English, and had a child diagnosed with ASD and no history of genetically-based conditions associated with ASD, such as fragile X syndrome. We made every effort to recruit intact families, but this was not always possible, given that both parents did not always attend school in Iowa, or in some cases one parent was not available to participate. Of the 139 parents of individuals with ASD included, there were 45 couples (i.e., 90 individuals), and 49 parents were from different families where only one parent was included. From this parent group, we were able to enroll 63 children with ASD. The remaining children were unable to participate due to different recruitment and testing restrictions making direct assessment unfeasible (e.g., living apart from parents).

Although the primary focus of this study concerned associations between parents’ childhood phenotypes and endophenotypes in parents in adulthood, along with ASD symptomatology in these parents’ children who were diagnosed with ASD, we also included a group of controls from Iowa in order to assess whether profiles of development in the ASD parent group showed any notable differences from individuals who did not go on to have a child with ASD, and also compared groups’ profiles against norms. Controls were recruited through referrals from existing studies at the University of Iowa, and through community advertising. Using the Autism Family History Interview (Bolton et al. 1994), controls were screened for personal and family history of ASD or related developmental disorders, as well as for language and cognitive delays to ensure typical development. Seventeen controls were parents of a child with typical development. Eleven controls were not parents, but were retained based on absence of any cases of autism, or language or cognitive delays in their extended family history. Groups were similar in IQ [t(86) = −.83, p = .41] in adulthood, but controls were significantly younger [t(165) = 5.17, p < .001, mean difference = 8.49 years] at the time of study enrollment. Full participant characteristics are presented in Table 1.

**Table 1:** Participant characteristics

	Parents of individuals with ASD	Control participants	Individuals with ASD
*N*	139	28	63
Sex (F:M)	79:60	17:11	9:54
Age [*M* (*SD*) Range]	46.5 (7.1) 25–65	38.1 (9.9) 22–60	14.8 (5.8) 2.6–28.2
IQ^a, b^ [*M* (*SD*) Range]	111.0 (12.2) 76–136	113.3 (12.3) 85–136	90.9 (23.3) 36–123
VIQ^b^ [*M* (*SD*) Range]	109.3 (13.0) 69–132	109.7 (14.8) 82–138	95.3 (16.8) 60–119
PIQ^b^ [*M* (*SD*) Range]	110.3 (12.0) 77–137	113.0 (14.1) 89–148	94.9 (24.5) 36–136
ADI-R algorithm scores			
Communication-verbal [*M*(*SD*) Range]			16.65 (4.60) 4–24
Communication-nonverbal [*M*(*SD*) Range]			12.40 (0.55) 12–13
Social reciprocity [*M*(*SD*) Range]			21.94 (5.89) 9–30
Restricted/repetitive behavior [*M*(*SD*) Range]			6.41 (2.38) 2–11
ADOS symptom severity [*M*(*SD*) Range]			7.17 (2.26) 1–10

^a^IQ was assessed in 93 parents of individuals with ASD who were able to participate in direct assessments, and in all controls

^b^IQ, verbal IQ (VIQ) and performance IQ (PIQ) measured with Wechsler abbreviated scale of intelligence (WASI), Wechsler intelligence scale for children (WISC) or Leiter-brief IQ

ADI-R was administered to parents of 56 indivdiuals with ASD, and ADOS was administered to 47 individuals with ASD.

### Procedures

Archival academic records were obtained for parents and controls. Participants also completed clinical-behavioral assessments, described below. Procedures were approved by University Institutional Review Boards. Informed consent was obtained by all participants.

### Parent Assessments

#### Parents’ Childhood Academic Performance

Academic testing scores were obtained from the Iowa Tests of Basic Skills (ITBS; Hoover et al. 2001) and its analog used in high school, the Iowa Tests of Educational Development (ITED; Forsyth et al. 2001). The ITBS and ITED are nationally standardized, norm-referenced tests that have been administered annually to Iowa school children since the 1950s (Hoover et al. 2001). The ITBS (administered in grades K-8) and ITED (grades 9–12) (hereafter referred to simply as the ITBS) were developed at the University of Iowa. Childhood testing records from all grades available (K-12) were obtained from the Iowa Testing Programs at the University of Iowa with participants’ consent. These tests assess core skills in language, reading, and math each year. Examples of language, reading, and math subtests with representative items are presented in Table 2.

**Table 2:** Description of subtests for language, reading, and math composite scales of the ITBS

Composite test	Subtest	Description
Language	Spelling	Identifying words spelled incorrectly from array of choices, assessing phonological and phonemic awareness
	Capitalization	Identifying capitalization errors in a body of text, including recognition of different lexical and syntactic categories denoted by capitalization rules
	Punctuation	Identifying punctuation errors (including under- and over-punctuation) in passages, identifying possessives, plurals, use of contractions, marking of compound and complex sentences, use of ellipsis
	Usage and expression	Identifying grammatical errors in a passage, such as verbs, pronouns, modifiers, agreement, etc., also involving judging discourse organization, clarity, and appropriateness of expression
Reading	Vocabulary	General vocabulary content assessed by matching words with correct pictures and completing sentences with appropriate word
	Reading comprehension	Assesses comprehension of sentences, passages, and stories, including drawing inferences to generalize about material
Math	Concepts	Assesses understanding of number properties, operations, numerical and geometric patterns, and measurement
	Problem solving	Solving word problems and interpreting data from graphs and tables

Over the time span that parents were administered the ITBS, there have been some minor changes in forms and subtest definition

#### Personality and Language Features of the BAP

Socially aloof and rigid personality features of the BAP were assessed with the Modified Personality Assessment Schedule (MPAS; Tyrer 1988). The M-PAS is a semi-structured interview that probes for the presence of subtle personality traits that are thought to mirror the core social and restricted/repetitive domains of ASD, and has been used extensively in prior studies of the BAP (Losh et al. 2008; Piven et al. 1997a, b). Subject interviews were consensus coded from video by two independent raters, with coding based on concrete examples of trait endorsement. M-PAS data were only collected with the ASD parent group, given the study focus on defining phenotypic associations within families of individuals with ASD. Thirty-nine percent of the ASD parent group was rated as positive for the social BAP, and 28% were positive for the rigid BAP. These rates are roughly comparable to those reported in prior work (e.g., Piven et al. 1997a, b).

The Pragmatic Rating Scale (PRS; Landa et al. 1992) was used to assess pragmatic language in parents. The PRS was coded from a 20-min semi-structured conversational interview, in which participants responded to conversational probes about their own lives with an examiner who was trained to elicit conversation by commenting and asking follow-up questions to tap various pragmatic skills. The PRS has been used extensively in studies of the BAP (Landa et al. 1992; Losh et al. 2008; Piven et al. 1997b), and consists of 25 operationally-defined items assessing the quality and content of conversational language (e.g., refraining from tangential speech, being neither too vague nor too detailed in conversational contributions, etc.). Two blinded independent raters coded each language sample, and consensus scores were determined through discussion. Parents of individuals with ASD averaged a PRS score of 9.95 (SD = 6.65), and controls averaged 5.96 (SD = 4.5). Accounting for minor differences in the PRS over time, these rates are comparable to scores reported in prior studies (e.g., Piven et al. 1997a, b; Losh et al. 2008).

#### Social Cognition

The Reading the Mind in the Eyes Test-Revised Version (Baron-Cohen et al. 2001) was administered as a measure of advanced social cognition. The Eyes Test requires participants to infer complex cognitive and emotional states based on viewing only the eye region of the face, with 4 potential answers.

### Assessment for Children with ASD

All children with ASD had received a clinical diagnosis of ASD using the DSM IV or DSM 5, and confirmed by medical records. Whenever possible diagnoses were also informed by gold standard diagnostic instruments, the Autism Diagnostic Interview-Revised (ADI-R) (Lord et al. 1994) or the Autism Diagnostic Observation Schedule (ADOS) (Lord et al. 2000). Raw scores of the ADI-R were summed for the diagnostic algorithms (which focus on behaviors at 4–5 years of age) to create summary scores for the overall diagnostic score, and for social, communication, and restricted/repetitive domains.

### Data Analysis

Estimates of academic skills and development over time were determined using hierarchical linear models (HLM; Raudenbush and Bryk 2002; Singer and Willett 2003). HLM allows for the estimation of fixed and random effects for model parameters. The fixed effects are the parameters for the sample as a whole, while the random effects are the parameters for each individual. The random effects were used to estimate and control the non-independence in the model arising from the clustering of data (Burchinal et al. 2006); in this case, the clustering of data within participant, resulting from their repeated measurement. Standard regression diagnostics were produced for primary HLM analyses (e.g., residuals plots, normal q–q plots) to screen that model assumptions were met. No violations of normality or heteroscadicity assumptions were evident. Random effects provide values for individual intercepts (i.e., performance, centered at third grade due to sparse data in earlier grades) and slopes (i.e., change over time). For a portion of families, data were available for both parents in a family, giving rise to non-independence for those data. HLM were run including random effects to account for the nesting within families. In all cases, the relevant family level effects were all essentially zero, suggesting that the within family nesting was not a significant source of variance and did not impact the model. To help control Type I error rates, interactions were not interpreted unless significant at p < .05. When interactions were not significant, only main effects are reported. For all analyses, p values are two-tailed.

In order to compare academic achievement and development between groups, a series of models were conducted for each of the test composites: language, reading, and math. Each analysis included group (ASD parent versus control), time, and their interaction as predictors of achievement on the ITBS. The dependent variables were scaled to indicate grade level. Due to sparse data in grades K-2, data were centered at third grade, and therefore group effects can be interpreted as the separation of the groups at that time point. A one-grade change per year was expected for each of the ITBS domains, represented by slope. HLM estimates of individual intercepts and slopes were exported for correlations examining relationships between parents’ early academic profiles and phenotypes in adulthood, as well as clinical symptom severity in parents’ children with ASD.

## Results

Both the ASD parent group and controls performed significantly above grade level in each domain overall (all *t* values >4.90, ps < .0001). However, the ASD parent group performed significantly lower than controls on the language composite, and showed marginally significant differences in reading and math. They also showed a significantly slower rate of growth in math, and marginally significantly slower growth in language and reading (See Table 3).

**Table 3:** Language, reading, and math composite performance across grades and groups

	ITBS composite score		
	Language	Reading	Math
Group and grade comparisons, fixed effects			
Grade	1.06 (0.04) p < .0001	1.08 (0.03) p < .0001	1.11 (0.04) p < .0001
Group	0.74 (0.29) p = .01	0.50 (0.28) p = .08	0.51 (0.27) p = .05
Grade*group	0.12 (0.07) p = .10	0.09 (0.05) p = .11	0.20 (0.08) p = .01

*Grade* is the amount of change expected each year. *Group* indicates the mean difference between groups. Grade*Group is the difference in the rate of change between groups

On subtests in the language domain, the ASD parent group scored significantly lower than controls in Punctuation and Language Usage, and showed significantly slower growth in Language Usage. Slower growth was also noted in the Capitalization subtest. In the reading domain, no group differences on subtests were noted, but the ASD parent group showed slower growth in the Comprehension subtest. Finally, in the math domain, the ASD parent group showed lower performance and slower growth in the Problem Solving subtest, and slower growth in Math Concepts (see Table 4).

**Table 4:** Performance on ITBS subtests across grades and groups

	ITBS subtest							
	Language				Reading		Math	
	Spelling	Capitalization	Punctuation	Usage	Vocabulary	Comprehension	Concepts	Problem solving
Fixed effects								
Grade	1.03 (0.04)^‡^	1.18 (0.05)^‡^	1.09 (0.05)^‡^	1.11 (0.05)^‡^	1.07 (0.03)^‡^	1.08 (0.03)^‡^	1.15 (0.04)^‡^	1.15 (0.04)^‡^
Group	0.44 (0.32)	0.26 (0.31)	0.77 (0.31)*	0.74 (0.33)*	0.51 (0.27)	0.49 (0.31)	0.42 (0.22)	0.61 (0.22)^†^
Grade*group	0.07 (0.08)	0.33 (0.1)^‡^	0.15 (0.1)	0.25 (0.1)*	0.05 (0.07)	0.12 (0.06)*	0.22 (0.07)^†^	0.18 (0.09)*

Notes: All Random Effects are significant. *p < .05, ^†^p < .01, ^‡^p < .001

### Associations Between Parents’ Childhood ITBS Performance and ASD Endophenotypes in Adulthood

A fractionated pattern of childhood development across domains, with more rapid growth in some domains and more static growth in others, was associated with the presence of the socially aloof trait of the BAP in adulthood in parents of individuals with ASD (r = .26, p < .05). There were no associations with the rigid dimension of the BAP, but the pragmatic language feature of the BAP was associated with relatively lower childhood language and reading scores (but not math) in the ASD parent group (r = −0.38, p < .001; r = −0.32, p < .01, respectively). Finally, performance on the Eyes Test of social cognition was associated with performance across domains in the ASD parent group (language r = .30, p < .01; math r = .28, p < .05; reading r = .41, p < .001) as well as with the rate of development in the language domain (r = .32, p < .01). No associations were detected between ITBS performance and pragmatic language in controls; however, controls’ performance on the Eyes Test was positively related to overall performance in the reading domain (r = .46, p < .05), but no other ITBS scales. Correlations with ITBS subscales followed the same general pattern as for the composite variables (see Table 5).

**Table 5:** ITBS subtests associated with ASD endophenotypes in parents

Group	Index of current functioning	Average spelling	Rate of development of spelling	Average capitalization	Rate of development of capitalization	Average punctuation	Rate of development of punctuation	Average usage	Rate of development of usage	Average vocabulary	Rate of development of vocabulary	Average reading comprehension	Rate of development of comprehension	Average concepts	Rate of development of concepts	Average problem solving	Rate of development of problem solving
ASD parents	BAP trait																
	Pragmatic language	−0.22	−0.21	−0.34^†^	−0.16	−0.47^‡^	−0.20	−0.32^†^	−0.25*	−0.35^†^	0.03	−0.28*	−0.09	−0.32*	−0.09	−0.32*	−0.17
	Social BAP	−0.07	−0.18	−0.10	−0.07	−0.17	−0.19	−0.02	−0.01	0.05	0.11	−0.01	0.00	−0.10	−0.04	−0.14	−0.10
	Rigid BAP	−0.07	−0.07	−0.07	0.03	0.07	0.05	0.01	−0.07	0.02	−0.14	−0.12	−0.03	0.04	−0.12	−0.01	−0.08
	Social cognition	0.27*	0.25*	0.24*	0.30*	0.30^†^	0.37^†^	0.22	0.25*	0.38^‡^	0.24*	0.40^‡^	0.18	0.38^‡^	0.32^†^	0.28*	0.32^†^
Controls	BAP trait																
	Pragmatic language	−0.13	−0.20	−0.17	−0.41*	−0.13	−0.39	−0.37	−0.14	−0.17	−0.24	−0.27	0.0	−0.23	−0.01	−0.26	−0.24
	Social cognition	0.05	0.22	0.08	0.027	0.16	0.31	0.40*	0.48*	0.46*	0.28	0.43*	0.02	0.18	0.29	0.31	0.27

*p < .05, ^†^p < .01, ^‡^p < .005

### Associations Between Parents’ Childhood ITBS and ASD Symptom Severity in Children

**Fig. 1:**
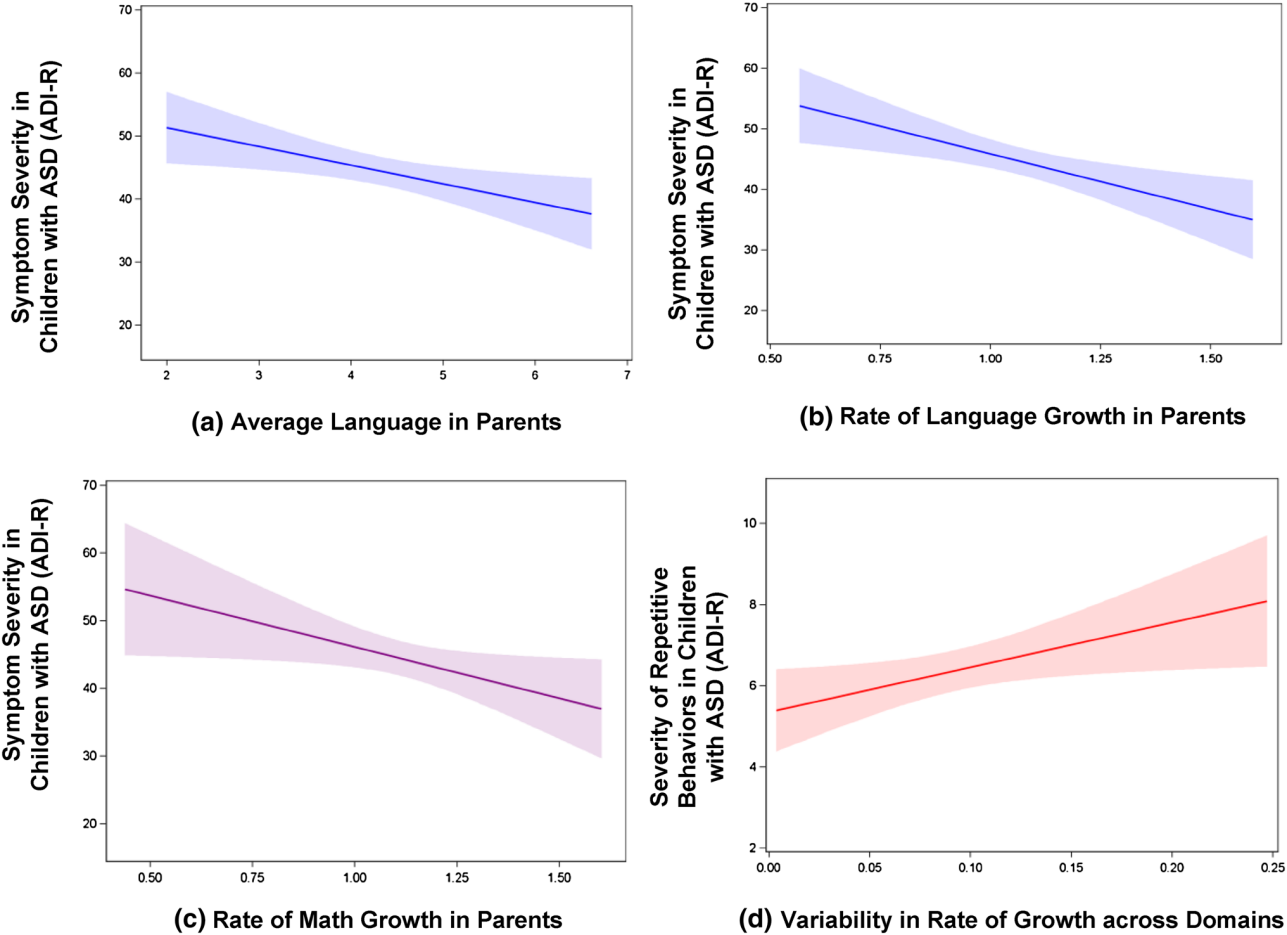
Patterns of childhood development on ITBS language and math among parents of individuals with ASD predict levels of symptom severity in children with ASD. Lower average language (**a**), and slower rates of language (**b**) and math (**c**) predicted higher symptom severity in children with ASD, and **d** more variable rates of development across domains (indexed by standard deviations of growth slopes) were related to increased severity of repetitive behaviors in children with ASD

Parents’ childhood ITBS performance was significantly associated with clinical symptom severity in children who were diagnosed with ASD (see Table 6). As illustrated in Fig. 1, relatively lower language performance, and slower rates of development in language and math in parents were associated with more severe ASD symptoms in children. These relationships were largely consistent across the subscales of language and math as well, with ASD symptom severity correlating particularly robustly with the rate of development in the language subtests of Capitalization, Punctuation, and Usage (see Table 7). Additionally, a fractionated rate of development across domains in parents was associated with increased severity of repetitive behaviors among children with ASD.

**Table 6:** Correlations between parents’ childhood ITBS language, reading, and math composite scores and their children’s ASD symptoms

	Childhood parental phenotypes						
	Average language ability	Language development rate	Average reading ability	Reading development rate	Average math ability	Math development rate	Variability in rates of development
ASD symptoms in children							
Overall symptoms	−0.28*	−0.30^†^	−0.16	−0.18	−0.20	−0.26*	−0.09
Social	−0.24*	−0.34^†^	−0.17	−0.15	−0.22	−0.27*	0.08
Communication	−0.24*	−0.26*	−0.13	−0.20	−0.16	−0.17	0.00
Restricted/repetitive	−0.27*	−0.34^†^	−0.17	−0.22^a^	−0.16	−0.30*	0.24*

ASD symptoms were assessed using the ADI-R (diagnostic algorithm). *p < .05, ^†^p < .01; ^a^p = 0.05

**Table 7:** Correlations between parents’ childhood ITBS subscale performance and their children’s ASD symptoms

	Average spelling	Rate of development of spelling	Average capitalization	Rate of development of capitalization	Average punctuation	Rate of development of punctuation	Average usage	Rate of development of usage	Average vocabulary	Rate of development of vocabulary	Average reading comprehension	Rate of development of comprehension	Average concepts	Rate of development of concepts	Average problem solving	Rate of development of problem solving
ADI-R																
Total score	−0.28*	−0.17	−0.20	−0.37^‡^	−0.21	−0.32^†^	−0.32^†^	−0.26*	−0.11	−0.17	−0.20	−0.17	−0.22*	−0.26*	−0.18	−0.21
Social	−0.22*	−0.15	−0.19	−0.37^‡^	−0.19	−0.33^†^	−0.30^†^	−0.25*	−0.12	−0.13	−0.21	−0.15	−0.22	−0.26*	−0.24*	−0.26*
Communication	−0.23*	−0.12	−0.14	−0.31^†^	−0.17	−0.24*	−0.27*	−0.20	−0.086	−0.20	−0.16	−0.16	−0.23*	−0.17	−0.08	−0.10
Restricted/ repetitive	−0.28*	−0.24*	−0.21	−0.34^†^	−0.20	−0.29^†^	−0.31^†^	−0.28*	−0.15	−0.18	−0.18	−0.22	−0.07	−0.33^†^	−0.16	−0.19

*p < .05, ^†^p < .01, ^‡^p < .001

Models detected no group differences between mothers and fathers, and no differences in parent–child correlations were detected. Although only 17 families were multiplex, we expanded mixed models to examine interactions with multiplex or simplex family status, and found no significant interactions with group or change over time. Patterns of association between parent and child characteristics, and parent childhood and adulthood characteristics were similar between multiplex and simplex families.

## Discussion

Making use of retrospective, longitudinal academic data, this study investigated childhood development in parents of individuals with ASD across the academic domains of language, reading, and math, with the goal of examining childhood profiles in parents that may consititute developmental endophenotypes reflecting genetic liability to ASD. Selected differences from controls were observed in ITBS performance, though they were quite subtle, with both groups performing above grade level overall. This is consistent with evidence that features of the BAP are subclinical in nature, do not involve functional impairment, and are observed in only a subset of parents (Losh et al. 2010, 2012; Martin et al. 2010; Piven et al. 1997b).

More significant were findings that childhood ITBS profiles predicted ASD endophenotypes in adulthood (social and language features of the BAP, and social cognition), and clinical symptom severity in children with ASD. These findings contribute to a substantial body of literature documenting a constellation of phenotypes occurring in parents that may reflect genetic liability to ASD, or the BAP, and associated neuropsychological traits (Adolphs et al. 2008; Billeci et al. 2016; Losh et al. 2009, 2010; Losh and Piven 2007). By characterizing associated phenotypic markers in childhood, before individuals’ status as a parent of a child with ASD could be known, findings may shed light on the developmental precursors of the BAP and provide measurable phenotypes in childhood that could potentially be targeted in family- and molecular-genetic studies of ASD, where such putative endophenotypes in relatives can help to identify more etiologically homogeneous subgroups.

Whereas differences from controls were subtle, the language domain seemed to stand out in differentiating the ASD parent group’s performance from that of controls who were screened for family and personal history of ASD and developmental delays. Together with a fractionated, or uneven, rate of development across domains, the ASD parent group’s performance on language-related tests was also notable in most robustly predicting features of the BAP in adulthood and ASD symptom severity in children. The ASD parent group displayed consistent differences across language subtests, with relatively lower scores and slower development in the language composite subtest of Language Usage, lower scores in Punctuation across grades, and slower growth on the Capitalization subtest over time. Performance in these subtests, and rate of growth over time in particular, also stood out as strong correlates of symptom severity in children with ASD, and of features of the BAP in adulthood.

The skills tapped by these subtests involve important aspects of grammatical language ability. For example, the Language Usage subtest examines the ability to detect grammatical errors in extended passages, as well as judgments of discourse organization and clarity. The Punctuation and Capitalization subtests tap more detailed grammatical knowledge required for integrating different sentential elements within and across utterances (e.g., differentiating declarative from interrogative statements and marking breaks and pauses across and within sentences with different punctuation; distinguishing personal pronouns and proper versus common nouns through capitalization differences). It is perhaps also of note that both math subtests on which differences were noted (Math Problem Solving and Concepts) and for which associations with child symptom severity also emerged, are relatively heavy in language demands, involving interpretation of lengthy and densely composed word problems. It may be that subtle differences in language related skills in childhood constitute a genetically meaningful phenotype evident in a subgroup of families. The significance of the language domain to ASD genetics is supported by the identification of several risk loci associated specifically with language phenotypes in ASD (Bartlett et al. 2014) along with the strong overlap of ASD and specific language impairment (SLI), where overlapping behavioral and neurocognitive phentoypes have been noted in both ASD and SLI, together with identification of shared language phenotyes in unaffected parents (Ruser et al. 2007; Tager-Flusberg 2016).

Knowledge of such childhood expressions of latent liability in patterns of academic performance across major curricular domains of language, reading, and math (and perhaps language in particular), a generation removed from affected individuals, may help to advance neural and genetic research by stratifying individuals and families to examine biological factors differentially associated with genetically meaningful developmental phenotypes in parents. The use of data from commonly administered standardized tests in this way could be rapidly translated to large scale studies where they may afford increased power and sensitivity for detecting gene–behavior relationships. To this end, recent work examining a wide range of phenotypes in a large sample of adolescents found that academic variables cosegregated significantly with genome-wide polygenic scores (Krapohl et al. 2016). In ASD, such deep phenotyping in family members could be a powerful means to decompose complex traits into genetically meaningful features, or endophenotypes, that may penetrate diagnostic boundaries and offer more tractable targets for study than complex clinical syndromes (Insel 2014), and help to inform different etiologic pathways, perhaps leading ultimately to new knowledge of the pathogenetics of ASD (Parikshak et al. 2015).

A significant factor to consider in future work concerns the relative homogeneity of the participant groups included. Subjects were necessarily drawn exclusively from the state of Iowa due to restrictions on the availability of academic records. And, while we made a concerted effort to include a representative ASD parent sample, recruiting over an extended period of time in both rural and metropolitan areas, these findings should be replicated in larger, more diverse population samples. Further, our control sample was relatively small, and controls were screened for personal and family history of ASD and language and cognitive delays, resulting in a control group who would be expected to have performed well on childhood academic testing. Although the most significant findings emerged from within-individual and within-family associations not impacted by the control sample, larger and more heterogeneous control groups should be included in future studies to replicate and determine the specificity of these findings, including whether associations between childhood academic performance and ASD endophenotypes may also exist in the general population.

In sum, by making use of valuable archival data, this study performed the first retrospective, longitudinal investigation of directly assessed childhood academic development in parents of individuals with ASD. Specific and early–emerging patterns of skill development were detected that appear to reflect genetic liability to ASD. These childhood phenotypes in parents appear very subtly expressed but were predictive of ASD endophenotypes in adulthood, and patterns of ASD symptom severity in children with ASD, and may therefore hold utility for studies of the cognitive and biological basis of ASD.

## References

[CR1] Adolphs R, Spezio ML, Parlier M, Piven J (2008). Distinct face-processing strategies in parents of autistic children. Current Biology: CB.

[CR2] American Psychiatric Association (2013). Diagnostic and statistical manual of mental disorders: DSM-5.

[CR3] Baron-Cohen S, Ring H, Chitnis X, Wheelwright S, Gregory L, Williams S, Bullmore E (2006). fMRI of parents of children with Asperger Syndrome: A pilot study. Brain and Cognition.

[CR4] Baron-Cohen S, Wheelwright S, Hill J, Raste Y, Plumb I (2001). The “reading the mind in the eyes” test revised version: A study with normal adults, and adults with Asperger syndrome or high-functioning autism. The Journal of Child Psychology and Psychiatry and Allied Disciplines.

[CR5] Baron-Cohen S, Wheelwright S, Jolliffe T (1997). Is there are a ‘language of the eyes’? Evidence from normal adults and adults with autism or Asperger Syndrome. Visual Cognition.

[CR6] Bartlett CW, Hou L, Flax JF, Hare A, Cheong SY, Fermano Z, Brzustowicz LM (2014). A genome scan for loci shared by autism spectrum disorder and language impairment. American Journal of Psychiatry.

[CR7] Bernier R, Gerdts J, Munson J, Dawson G, Estes A (2011). Evidence for broader autism phenotype characteristics in parents from multiple-incidence autism families. Autism Research.

[CR8] Billeci L, Calderoni S, Conti E, Gesi C, Carmassi C, Dell’Osso L, Guzzetta A (2016). The broad autism (endo)phenotype: Neurostructural and neurofunctional correlates in parents of individuals with autism spectrum disorders. Frontiers in Neuroscience.

[CR9] Bolton P, Macdonald H, Pickles A, Rios PA, Goode S, Crowson M, Rutter M (1994). A case-control family history study of autism. Journal of Child Psychology and Psychiatry.

[CR10] Burchinal M, Nelson L, Poe M (2006). Growth curve analysis: An introduction to various methods for analyzing longitudinal data. Monographs of the Society for Research in Child Development.

[CR11] Forsyth RA, Ansley TN, Feldt LS, Alnot SD (2001). Iowa Tests of Educational Development.

[CR12] Gottesman II, Gould TD (2003). The endophenotype concept in psychiatry: Etymology and strategic intentions. The American Journal of Psychiatry.

[CR13] Hogan-Brown AL, Hoedemaker RS, Gordon PC, Losh M (2014). Eye-voice span during rapid automatized naming: Evidence of reduced automaticity in individuals with autism spectrum disorder and their siblings. Journal of Neurodevelopmental Disorders.

[CR14] Hoover H, Dunbar S, Frisbie D (2001). Iowa Tests of Basic Skills, Form A.

[CR15] Insel TR (2014). The NIMH Research Domain Criteria (RDoC) Project: precision medicine for psychiatry. American Journal of Psychiatry.

[CR16] Investigators DDMNSYP (2014). Prevalence of autism spectrum disorder among children aged 8 years—Autism and developmental disabilities monitoring network, 11 sites, United States, 2010. MMWR Surveillance Summaries.

[CR17] Krapohl E, Euesden J, Zabaneh D, Pingault JB, Rimfeld K, von Stumm S, Plomin R (2016). Phenome-wide analysis of genome-wide polygenic scores. Molecular Psychiatry.

[CR18] Landa R, Folstein SE, Isaacs C (1991). Spontaneous narrative-discourse performance of parents of autistic individuals. Journal of Speech and Hearing Research.

[CR19] Landa R, Piven J, Wzorek MM, Gayle JO, Chase GA, Folstein SE (1992). Social language use in parents of autistic individuals. Psychological Medicine.

[CR20] Lord C, Risi S, Lambrecht L, Cook EH, Leventhal BL, DiLavore PC, Rutter M (2000). The autism diagnostic observation schedule-generic: A standard measure of social and communication deficits associated with the spectrum of autism. Journal of Autism and Developmental Disorders.

[CR21] Lord C, Rutter M, Le Couteur A (1994). Autism diagnostic interview-revised: A revised version of a diagnostic interview for caregivers of individuals with possible pervasive developmental disorders. Journal of Autism and Developmental Disorders.

[CR22] Losh M, Adolphs R, Piven J, Dawson G, Amaral D, Geschwind D (2011). The broad autism phenotype. Autism spectrum disorders.

[CR23] Losh M, Adolphs R, Poe MD, Couture S, Penn D, Baranek GT, Piven J (2009). Neuropsychological profile of autism and the broad autism phenotype. Archives of General Psychiatry.

[CR24] Losh M, Childress D, Lam K, Piven J (2008). Defining key features of the broad autism phenotype: A comparison across parents of multiple- and single-incidence autism families. American Journal of Medical Genetics Part B: Neuropsychiatric Genetics.

[CR25] Losh M, Esserman D, Piven J (2010). Rapid automatized naming as an index of genetic liability to autism. Journal of Neurodevelopmental Disorders.

[CR26] Losh M, Klusek J, Martin GE, Sideris J, Parlier M, Piven J (2012). Defining genetically meaningful language and personality traits in relatives of individuals with fragile X syndrome and relatives of individuals with autism. American Journal of Medical Genetics Part B: Neuropsychiatric Genetics.

[CR27] Losh M, Piven J (2007). Social-cognition and the broad autism phenotype: Identifying genetically meaningful phenotypes. Journal of Child Psychology and Psychiatry.

[CR28] Lowe JK, Werling DM, Constantino JN, Cantor RM, Geschwind DH (2015). Social responsiveness, an autism endophenotype: Genomewide significant linkage to two regions on chromosome 8. The American Journal of Psychiatry.

[CR29] Martin, G. E., Losh, M., Klusek, J., & Harris, A. (2010). *Pragmatic language and social cognitive overlap in children with autism and fragile X syndrome*. Paper presented at the International Meeting for Autism Research, Philadelphia, PA.

[CR30] Murphy M, Bolton P, Pickles A, Fombonne E, Piven J, Rutter M (2000). Personality traits of the relatives of autistic probands. Psychological Medicine.

[CR31] Norton ES, Wolf M (2012). Rapid automatized naming (RAN) and reading fluency: Implications for understanding and treatment of reading disabilities. Annual review of psychology.

[CR32] Ozonoff S, Young GS, Belding A, Hill M, Hill A, Hutman T, Iosif A-M (2014). The broader autism phenotype in infancy: When does it emerge?. Journal of the American Academy of Child and Adolescent Psychiatry.

[CR33] Parikshak NN, Gandal MJ, Geschwind DH (2015). Systems biology and gene networks in neurodevelopmental and neurodegenerative disorders. Nature Reviews Genetics.

[CR34] Pilowsky T, Yirmiya N, Gross-Tsur V (2007). Neuropsychological functioning of siblings of children with autism, siblings of children with mental retardation of unknown genetic etiology. Journal of Autism and Developmental Disorders.

[CR35] Piven, J., Palmer, P., Jacobi, D., Childress, D., & Arndt, S. (1997a). Broader autism phenotype: Evidence from a family history study of multiple-incidence autism families. *American Journal of Psychiatry, 154*(2), 185–190. 10.1176/ajp.154.2.185 9016266

[CR36] Piven, J., Palmer, P., Landa, R., Santangelo, S., Jacobi, D., & Childress, D. (1997b). Personality and language characteristics in parents from multiple-incidence autism families. *American Journal of Medical Genetics Part B: Neuropsychiatric Genetics, 74*(4), 398–411. 9259376

[CR37] Piven J, Wzorek M, Landa R, Lainhart J, Bolton P, Chase GA, Folstein S (1994). Personality characteristics of the parents of autistic individuals. Psychological Medicine.

[CR38] Raudenbush, S. W., & Bryk, A. S. (2002). *Hierarchial linear models: Applications and data analysis methods*. Thousand Oaks, CA: Sage Publications.

[CR39] Ruser TF, Arin D, Dowd M, Putnam S, Winklosky B, Rosen-Sheidley B, Folstein S (2007). Communicative competence in parents of children with autism and parents of children with specific language impairment. Journal of Autism and Developmental Disorders.

[CR40] Singer, J. D., & Willett, J. B. (2003). *Applied longitudinal data analysis: Modeling change and event occurrence*. New York: Oxford University Press.

[CR41] Tager-Flusberg H (2016). Risk factors associated with language in autism spectrum disorder: Clues to underlying mechanisms. Journal of Speech Language and Hearing Research.

[CR42] Tick B, Bolton P, Happé F, Rutter M, Rijsdijk F (2015). Heritability of autism spectrum disorders: A meta-analysis of twin studies. Journal of Child Psychology and Psychiatry.

[CR43] Tyrer P, Tyrer P (1988). Personality assessment schedule. Personality disorders: Diagnosis, management, and course.

[CR44] Virkud YV, Todd RD, Abbacchi AM, Zhang Y, Constantino JN (2009). Familial aggregation of quantitative autistic traits in multiplex versus simplex autism. American Journal of Medical Genetics Part B: Neuropsychiatric Genetics.

